# The Additive Influence of Propane-1,2-Diol on SDS Micellar Structure and Properties

**DOI:** 10.3390/molecules26123773

**Published:** 2021-06-21

**Authors:** Martina Gudelj, Paola Šurina, Lucija Jurko, Ante Prkić, Perica Bošković

**Affiliations:** 1 Department of Chemistry, Faculty of Science, University of Split, Ruđera Boškovića 33, 21000 Split, Croatia; margud@pmfst.hr (M.G.); psurina@pmfst.hr (P.Š.); 2 Faculty of Mechanical Engineering, University of Maribor, Smetanova 17, 20000 Maribor, Slovenia; luce.jurko@gmail.com; 3 Department of Chemistry, Faculty of Chemistry and Technology, University of Split, Ruđera Boškovića 35, 21000 Split, Croatia; prkic@ktf-split.hr

**Keywords:** micellar structure, sodium dodecyl sulfate, propane-1,2-diol, conductivity, hydrodynamic size, ^1^H NMR

## Abstract

Micellar systems are colloids with significant properties for pharmaceutical and food applications. They can be used to formulate thermodynamically stable mixtures to solubilize hydrophobic food-related substances. Furthermore, micellar formation is a complex process in which a variety of intermolecular interactions determine the course of formation and most important are the hydrophobic and hydrophilic interactions between surfactant–solvent and solvent–solvent. Glycols are organic compounds that belong to the group of alcohols. Among them, propane-1,2-diol (PG) is a substance commonly used as a food additive or ingredient in many cosmetic and hygiene products. The nature of the additive influences the micellar structure and properties of sodium dodecyl sulfate (SDS). When increasing the mass fraction of propane-1,2-diol in binary mixtures, the *c.m.c.* values decrease because propane-1,2-diol is a polar solvent, which gives it the ability to form hydrogen bonds, decreasing the cohesivity of water and reducing the dielectric constant of the aqueous phase. The values of $\Delta G_{m}^{0}$ are negative in all mixed solvents according to the reduction in solvophobic interactions and increase in electrostatic interaction. With the rising concentration of cosolvent, the equilibrium between cosolvent in bulk solution and in the formed micelles is on the side of micelles, leading to the formation of micelles at a lower concentration with a small change in micellar size. According to the ^1^H NMR, with the addition of propylene glycol, there is a slight shift of SDS peaks towards lower ppm regions in comparison to the D_2_O peak. The shift is more evident with the increase in the amount of added propane-1,2-diol in comparison to the NMR spectra of pure SDS. Addition of propane-1,2-diol causes the upfield shift of the protons associated with hydrophilic groups, causing the shielding effect. This signifies that the alcohol is linked with the polar head groups of SDS due to its proximity to the SDS molecules.

## 1. Introduction

The micellization of surfactants has long been of interest to scientists around the world due to their wide application in various industries, especially cosmetics, pharmaceuticals, detergents, etc. [1,2]. In addition to the choice of surfactant to be used for specific purposes, the medium in which the micellization of this surfactant is studied is equally important.

The importance of testing in different solvents and solvent mixtures stems from the fact that the properties of the solvent/solvent mixture itself can significantly affect the micellization process [3,4,5,6,7,8,9,10]. In addition, the use of different solvents allows the formation of micelles under optimal conditions, thus increasing efficiency and reducing production costs, but also increasing environmental efficiency [1,11].

Micellar formation is a complex process in which a variety of intermolecular interactions determine the course of formation. Among the most important are the hydrophobic and hydrophilic interactions between surfactant–solvent and solvent–solvent [12,13].

Glycols are organic compounds that belong to the group of alcohols. They have two O-H groups and a hydrophobic carbon chain. Both parts of the molecule are capable of interacting with anionic surfactants when present as cosolvents in mixtures. Among them, propane-1,2-diol (PG) is a substance commonly used as a food additive or ingredient in many cosmetic and hygiene products. Additionally, it can dissolve some substances better than water and is also good at retaining moisture. This makes it very useful as a food additive, so it can be found in a wide variety of processed foods and drinks [14,15,16].

SDS is one of the most researched surfactants. In the middle of the 20th century, the study of its basic parameters in water as a solvent began [17]. Different methods were used to determine *c.m.c.* of SDS and other surfactants [18,19]. To date, the conductometric technique is one of the simplest and often used methods for determining *c.m.c.* of ionic surfactants [20,21]. Following the studies in water, many different solvents and mixed systems and their effects on *c.m.c.* and other thermodynamic parameters have been tested over the years [19,20,21,22]. In addition to mixed solvents, mixtures of SDS with other surfactants [23,24] and the behavior of SDS in known, previously tested solvents with the addition of certain salts have also been tested [20,21,25].

Sodium dodecyl sulfate (SDS) has been studied in various binary glycol–water mixtures [13,26,27], but its interactions with propane-1,2-diol have not yet been investigated.

In this work, we observe how the presence of propane-1,2-diol in a water–propane-1,2-diol mixture affects the micelle formation process of SDS. The formation of micelles is studied in solvent mixtures with different propane-1,2-diol mass fractions.

In addition to the influence of solvents, the influence of temperature on micelle formation is also investigated as well as the size and stability of formed micelles.

## 2. Results

### 2.1. Determination of c.m.c.

**Figure 1:**
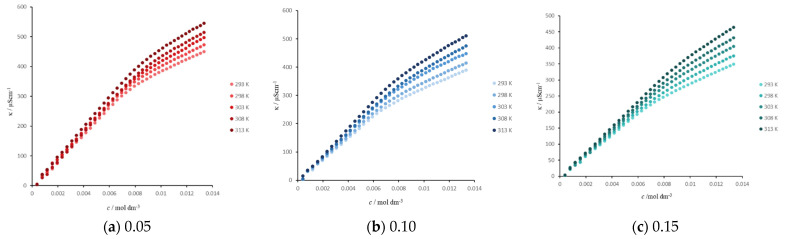
Plot of electrical conductivity vs. concentration for SDS in the binary mixture of water–propane-1,2-diol at different temperatures, where the mass fraction of propane-1,2-diol is equal to (**a**) 0.05, (**b**) 0.10 and (**c**) 0.15.

Electrical conductivity values of SDS (Figure 1) were measured at different mass fractions of propane-1,2-diol in water–propane-1,2-diol mixtures. The measured values of electrical conductivity were used to construct a plot of electrical conductivity vs. concentration. The intersection of the two plot lines representing the pre-micellar and post-micellar concentrations of SDS gives the *c.m.c* (Table 1).

#### Determination of Thermodynamic Parameters of SDS in Water–Propane-1,2-Diol

The thermodynamic parmeters of micellization-free energy ($\Delta G_{m}^{0}$), enthalpy ($\Delta H_{m}^{0}$) and entropy ($\Delta S_{m}^{0}$) (Table 2) were determined for SDS in binary mixtures of water–propane-1,2-diol with different mass fractions of propane-1,2-diol. The values of the electrical conductivity are shown in Figure 1. The Gibbs free energy was calculated from the equation:

(1) $$\Delta_{.}G_{m}^{0}=\left(2-\alpha \right)RT\ln\chi_{cmc}$$

where *R* represents a universal gass constant, *T* thermodynamic temperature and $\ln\chi_{cmc}$ is the natural logarithm of *c.m.c.* expressed as a mole fraction.

The change in enthalpy is calculated using the Gibbs–Helmholtz equation:

(2) $$\Delta H_{m}^{o}=-T^{2}\frac{\delta \left(\Delta G_{m}^{o}/T\right)}{\delta T}=-\left(2-\alpha \right)RT^{2}\frac{\delta \ln x_{c.m.c.}}{\delta T}$$

Plots of $\ln\chi_{cmc}$ vs. temperature were made and, fitting the plot with Equation (4), the first derivate was determined at each temperature and substituted into a Gibbs–Helmholtz Equation (2) [28].

(3) $$\ln x_{c.m.c.}=A_{o}+A_{1}\ln T$$

Enthropy and free energy values calculated in previous steps were used to determine entropy of micellization using the relation:

(4) $$\Delta S_{m}^{0}=\frac{\Delta H_{m}^{0}-\Delta_{.}G_{m}^{0}}{T}$$

The effect of propane-1,2-diol on the micellization process is calculated by the following equation:

(5) $$\Delta G_{trans}=\Delta G_{m\left(\mathrm{PG}+\mathrm{water}\right)}-\Delta G_{m\left(\mathrm{water}\right)}$$

In addition, the degree of dissociation of the counterion (α) was determined from the slope ratio of the lines above (*A*_2_) and below (*A*_1_) *c.m.c.* from the following equation:

(6) $$\alpha =\frac{A_{2}}{A_{1}}$$

### 2.2. Aggregation Size and Stability

**Figure 2:**
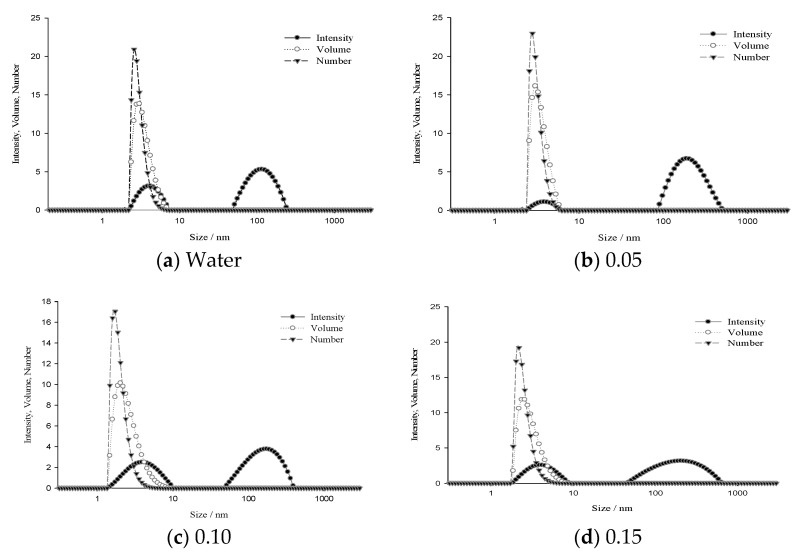
Dynamic light scattering (DLS) measurement of the size distribution of SDS (0.01 mol dm^−3^ SDS solution) in the binary mixture of water and (**a**) 0 mass fraction of propane-1,2-diol, (**b**) 0.05 mass fraction of propane-1,2-diol, (**c**) 0.10 mass fraction of propane-1,2-diol and (**d**) 0.15 mass fraction of propane-1,2-diol.

To study the aggregation behavior, the size distributions and stability of the aggregates formed by SDS in the binary mixture of water–propane-1,2-diol at the same concentration (0.01 mol dm^−3^ SDS solution) were investigated using dynamic light scattering (DLS) and zeta potential measurements (Figure 2 and Table 3).

### 2.3. H NMR Studies

**Figure 3:**
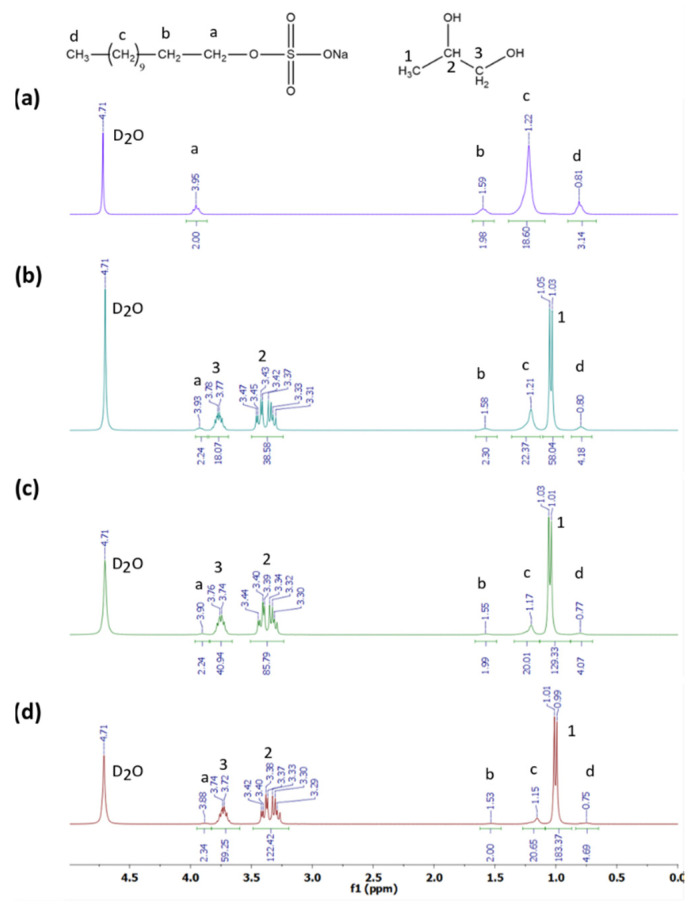
^1^H NMR results of SDS (0.01 mol dm^−3^ SDS solution) in the binary mixture of water and (**a**) 0 mass fraction of propane-1,2-diol, (**b**) 0.05 mass fraction of propane-1,2-diol, (**c**) 0.10 mass fraction of propane-1,2-diol and (**d**) 0.15 mass fraction of propane-1,2-diol.

Interactions of propane-1,2-diol with micellar aggregates in mixed solvents investigated by the ^1^H NMR technique are presented in Figure 3.

## 3. Discussion

The results of the study (Table 1) show that as the mass fraction of propane-1,2-diol in a binary mixtures increases, the *c.m.c.* values seem to decrease. A possible explanation for this observation lies in the nature of the cosolvent. Propane-1,2-diol is a polar solvent with two O-H groups, which gives it the ability to form hydrogen bonds and, consequently, its presence in mixtures decreases the cohesivity of water and reduces the dielectric constant of the aqueous phase. A similar effect was found in butane-1,2-diol–water mixtures as well as in ethane-1,2-diol–water mixtures. [13,26]. As a result, the repulsion between the ionic heads of the surfactant increases and the interaction between the hydrophobic tails is disturbed. The effect is more pronounced the higher the proportion of propane-1,2-diol is in the mixture. Another interesting effect is present at a 0.15 mass fraction of propane-1,2-diol. The main reason for the slight increase in *c.m.c.* compared to a lower mass fraction of cosolvent is that a decrease in the dielectric constant of the aqueous phase causes an increase in repulsion between the ionic head groups, thus opposing micellization.

The values of the degree of counterion dissociation in mixtures are higher than that in pure water. Additionally, with a larger proportion of glycol in a mixture, the increase becomes more pronounced. The values indicate that the cosolvent solubilization at the micellar surface reduces the charge density and causes the increase in the dissociation [13].

The effect of the addition of polar organic solvent on the micellization process has been quantitatively estimated from the standard Gibbs free energy of micellization $\left(\Delta G_{m}^{o}\right).$ The micellization process can be described by the equilibrium between surfactant monomers, counterions and monodisperse micelles. Table 2 summarizes the Gibbs free energy ($\Delta G_{m}^{o},\;\Delta G_{trans}^{o})$ values obtained using Equations (1) and (5) for a surfactant in different solvent mixures. The values of $\Delta G_{m}^{o}\;$ are negative in all mixed solvents and increase with increasing mass fraction of propane-1,2-diol. According to the reduction of the solvophobic interaction and increase in electrostatic interaction, the solubility of the hydrocarbon tail increases and the bulk phase becomes a good solvent for the surfactant. As a result, micelle formation becomes less favorable. Values of $\Delta G_{trans}^{o}\;$ were positive and increased with increasing mass fraction of propane-1,2-diol. The main reason is that the hydrophobic part of the surfactant is solvated by the hydrocarbon chain of the propane-1,2-diol and the hydrophilic part is solvated by the water molecules. The results are in agreement with the literature [5,13].

The enthalpy values are given in Table 2. The micellization process is exotermic (obtained from Equations (2) and (3)) in nature and the process becomes more exotermic with increasing cosolvent mass fraction and temperature due the possible interactions between surfactant–solvent and solvent–solvent molecules [13,26].

As the cosolvent content and temperature increase, the values of $\Delta S_{m}^{0}$ decrease, showing that the $\Delta H_{m}^{0}$ becomes a more dominant factor because of the reduction in the amount of water surrounding the hydrophobic part of the surfactant and the amount of water hydrogen-bonded upon micellar solubilization of the cosolvent. The increase in *c.m.c.* values is present in 0.15 mass fraction of propane-1,2-diol compared to other mixtures. This may be because the cosolvent is a water structure-breaking solute and at the threshold there are enough cosolvent molecules to break water–water bonds, decreasing the enthalpic contributions [29]. The micellization process is governed primarily by the entropy increase, because of the tendency of the hydrophobic group of the surfactant to transfer from the solvent to the interior of the micelle [29].

The size of the particles was calculated using the DLS instrument software according to the obtained correlation functions. For a solution with a 0.01 mol dm^−3^ concentration of SDS in water/mixed solvents, the result of the calculation according to the intensity of the scattered light corresponds to two types of spheres. The result of the calculation according to the volume and number is only one type of spherical particle with an average diameter of around 4 nm (listed in Table 3) which is related to the micellar colloid of SDS in water/mixed solvents, similar to literature values [30]. When transforming the intensity distribution to a volume and number distribution, the result only shows a single peak. The volume and number contribution from the second component is therefore so small (<0.01%) that it is no longer displayed. The reason for a reduction in the number of peaks is that the contribution is so small that it is no longer relevant in that transformation and discussion. According to the results from the rising concentration of cosolvent, equilibrium between cosolvent in bulk solution and in the formed micelles is on the side of micelles, leading to the formation of micelles at a lower concentration with a small change in micellar size. After a threshold (0.15 mass fraction of cosolvent), it can be found from Table 1, Table 2 and Table 3 that there is a decrease in *c.m.c.* values and the absolute value of zeta potential, and an increase in the effective degree of dissociation. A decrease in the charge density at the micellar surface due to the decrease in the size of the micelles causes an increase in the effective degree of dissociation at higher propane-1,2-diol concentrations.

According to the ^1^H NMR (Figure 3), SDS presents four peaks: (i) 3.95 ppm (a), which corresponds to a methylene group attached to the sulfate group; (ii) 1.59 ppm (b) and 1.22 ppm (c) peaks corresponding to the rest of the methylene groups of SDS and (iii) a 0.81 ppm (d) peak corresponding to the terminal methyl group. With the addition of propylene glycol, there is a slight shift of SDS peaks towards lower ppm regions in comparison to the D_2_O peak.

The shift is more evident with the increase in the amount of added propane-1,2-diol in comparison to the NMR spectra of pure SDS. The CH_3_ end group shows decreased ppm values from 0.80 ppm to 0.80, 0.77 and 0.75 with the increase in the mass ratio of propylene glycol, respectively. A similar trend is observed with (CH_2_)_9_ protons from 1.22 to 1.15 ppm, for β-CH_2_ from 1.59 to 1.53 ppm and α-CH_2_ from 3.95 to 3.98 ppm. The addition of propane-1,2-diol causes the upfield shift of the protons associated with hydrophilic groups causing the shielding effect. This signifies that the alcohol links with the polar head groups of SDS due to its proximity to the SDS molecules [31]. Similar behavior was described by Atanase et al., however, with the deshielding properties of applied copolymers with SDS [32,33].

The upfield shift of the proton peaks of propane-1,2-diol from 1.03–0.99 ppm, 3.43–3.38 ppm and 3.77–3.72 ppm is caused by insertion of the alkyl chain of propylene glycol within the SDS micelle, causing the additional shielding of the associated protons of the alcohol due to their small structure.

Although the NMR measurements were conducted at the same temperature, a decrease in peak intensity of SDS protons can be observed with the increase in the mass fraction of propylene glycol. According to the DLS measurements, there is an increase in the micellar diameter with the addition of 0.05 mass fraction of propane-1,2-diol. With an additional increase in the alcohol, the diameter decreases. This leads to a smaller micellar core and more compact structure of the micelle itself, and decreased mobility of the SDS protons [34].

Integration of the peaks showed the corresponding number of protons of methyl groups for SDS measurements. Compared to the other spectra, with the addition of propane-1,2-diol, integration values of the SDS proton peaks did not change significantly. Looking at the peak areas of propane-1,2-diol, there is a drastic change in the integral values which increase with the increase in the mass ratio. Compared to the number of protons of the lowest integral value of the peak at 3.7 ppm, and dividing with other integral values of peaks corresponding to propane-1,2-diol, there is always the same ratio of protons, which correspond to the empirical number of protons attached to the carbon atom of propane-1,2-diol, which is 1:2:3.

## 4. Materials and Methods

### 4.1. Chemicals

The reagents used in this research were propane-1,2-diol (Sigma-Aldrich St. Louis, USA, ACS reagent, purity ≥ 99.5%) and sodium dodecyl sulfate (Sigma-Aldrich, Sigma-Aldrich St. Louis, USA ACS reagent, purity ≥ 99.0%). The ultrapure water used in this study was treated with the Elga Purelab flex water purification device.

### 4.2. Conductivity Measurements

Conductivity values were measured at various concentrations of sodium dodecyl sulfate (SDS) in binary solvent mixtures of water–propane-1,2-diol. The electrical conductivity values were determined using the Mettler Toledo FiveEasy conductivity meter. The experimental procedure began by pouring 100 cm^3^ of ultrapure water into a glass reaction cell. The reaction cell was then hermetically closed by a Teflon lid and placed into a Brosan Ultratherm BWT-U (a constant temperature was maintained with a deviation of ±0.1 °C). After achievement of thermal equilibrium, the electrical conductivity was determined. Then, the 1 cm^3^ of 0.08 mol dm^−3^ surfactant solution was added to the cell using a micropipette and the electrical conductivity was read. The experiment finished after forty measurements. All experiments were conducted in triplicate.

### 4.3. Size and Stability

The size distribution and zeta potential of solutions were measured using a Litesizer 500 (Anton Paar, Graz, Austria) at 25 °C using cuvettes (for size and PDI) or capillary cells (for zeta potential). Before the measurements, each solution was filtered with a 0.2 μm PTFE membrane filter in order to remove dust. The size distribution and zeta potential were measured in 0.01 mol dm^−3^ surfactant solution and presented as the average value of three measurements.

### 4.4. H NMR Studies

^1^H NMR studies were performed with 300 MHz Bruker NMR machine. Samples were prepared by dissolving an exact amount of SDS into the solution of defined composition of propylene glycol and D_2_O (4.71 ppm). The chemical shifts are reported in δ (ppm) to TMS (0 ppm). Characterization of the signals: s = singlet, t = triplet, m = multiplet, ds = double singlet. Integration was determined as the relative number of atoms.

## 5. Conclusions

The chemical behavior of SDS in a binary solvent mixture consisting of water and propane-1,2-diol in different ratios at the temperature range 293–313 K was observed by electrical conductance, DLS and ^1^H NMR. When increasing the mass fraction of propane-1,2-diol in binary mixtures, the *c.m.c.* values seem to decrease. Propane-1,2-diol is a polar solvent with two O-H groups, which gives it the ability to form hydrogen bonds and, consequently, its presence in mixtures decreases the cohesivity of water and reduces the dielectric constant of the aqueous phase. The values of $\Delta G_{m}^{o}\;$ are negative in all mixed solvents according to the reduction of solvophobic interactions and the increase in electrostatic interaction, and the solubility of the hydrocrabon tail increases and the bulk phase becomes a good solvent for the surfactant. As a result, micelle formation becomes less favorable. As the cosolvent content and temperature increase, the values of $\Delta S_{m}^{0}$ decrease, showing that the $\Delta H_{m}^{0}$ becomes a more dominant factor because of a decrease in the amount of water surrounding the hydrophobic part of the surfactant and the amount of water hydrogen-bonded upon micellar solubilization of the cosolvent. With the rising concentration of the cosolvent, equilibrium between the cosolvent in bulk solution and in the formed micelles is on the side of micelles, leading to the formation of micelles at a lower concentration with a small change in micellar size. According to the ^1^H NMR, SDS presents four peaks. With the addition of propylene glycol, there is a slight shift of SDS peaks towards lower ppm regions in comparison to the D_2_O peak. The shift is more evident with the increase in the amount of added propane-1,2-diol in comparison to the NMR spectra of pure SDS.

Although the NMR measurements were conducted at the same temperature, a decrease in peak intensity of SDS protons can be observed with the increase in the mass ratio of propylene glycol. According to the DLS measurements, there is an increase in the micellar diameter with the addition of 0.05 mass fraction of propane-1,2-diol. With an additional increase in the alcohol, the diameter decreases. This leads to a smaller micellar core and more compact structure of the micelle itself, and decreased mobility of the SDS protons.

## Figures and Tables

**Table 1:** Values of *c.m.c.* of SDS in pure water and water–propane-1,2-diol mixtures where mass fraction of glycol component equals 0.05, 0.10 and 0.15.

Temperature (K)	*c.m.c.* Water * (mol dm^−3^)	*c.m.c.* (0.05) (mol dm^−3^)	*c.m.c.* (0.10) (mol dm^−3^)	*c.m.c.* (0.15) (mol dm^−3^)
293	8.48	7.64	7.01	7.35
298	8.38	7.74	7.15	7.72
303	8.31	7.79	7.74	7.73
308	8.39	7.83	7.98	8.22
313	8.46	8.61	8.12	8.50

* Ref. [13].

**Table 2:** Thermodynamic parameters for micellization of SDS in different mass fraction of propane-1,2-diol.

Mass Fraction	Temperature (K)	*α*	$\Delta G_{m}^{0}$ (kJ mol^−1^)	$\Delta H_{m}^{0}$ kJ mol^−^^1^	$\Delta S_{m}^{0}$ (J mol^−^^1^ K^−^^1^)	$\Delta G_{trans}^{0}$ (kJ mol^−1^)
Water *	293	0.34	−35.28	1.87	126.77	−
	298	0.36	−35.92	0.10	120.87	−
	303	0.35	−36.48	−1.66	114.92	−
	308	0.39	−37.05	−3.42	109.17	−
	313	0.39	−37.59	−5.19	103.52	−
0.05	293	0.47	−32.99	−5.57	93.58	2.29
	298	0.48	−33.29	−5.63	92.82	2.63
	303	0.53	−32.71	−5.53	89.69	3.77
	308	0.53	−33.23	−5.63	89.63	3.82
	313	0.52	−33.64	−5.75	89.08	3.95
0.10	293	0.53	−31.94	−8.79	79.00	3.34
	298	0.54	−32.06	−8.84	77.92	3.86
	303	0.54	−32.28	−8.98	76.90	4.20
	308	0.59	−31.59	−8.82	73.92	5.46
	313	0.59	−32.19	−9.01	74.06	5.40
0.15	293	0.58	−30.46	−7.26	79.17	4.82
	298	0.61	−30.17	−7.21	77.04	5.75
	303	0.63	−30.23	−7.25	75.85	6.25
	308	0.68	−29.40	−7.10	72.41	7.65
	313	0.70	−29.31	−7.10	70.95	8.28

* Ref. [13].

**Table 3:** Size distribution and zeta potential of SDS in the binary mixture of water–propane-1,2-diol at the same surfactant concentration (0.01 mol dm^−3^ SDS solution).

Mass Fraction	Zeta Potential/mV	Size/nm			
		Intensity Peaks		Volume Peak	Number Peak
		1.	2.		
**water**	−37.4 ± 16.2	4.210 ± 1.104	119.1 ± 43.00	3.46 ± 0.7105	3.012 ± 0.4651
**0.05**	−18.3 ± 8.2	4.605 ± 1.431	145.0 ± 63.37	3.388 ± 0.7672	2.832 ± 0.4606
**0.10**	−16.6 ± 7.40	4.251 ± 1.673	169.7 ± 83.58	2.602 ± 0.6933	1.995 ± 0.3877
**0.15**	−17.8 ± 9.49	4.273 ± 1.351	218.7 ± 128.8	3.887 ± 0.6844	2.529 ± 0.4447

## Data Availability

The original contributions generated for this study are included in the article.

## Sample Availability

Samples are not available from the authors.
